# Case Report: Herbal nutritional intervention in a critically ill ALS patient with multidrug-resistant infection

**DOI:** 10.3389/fmed.2026.1912856

**Published:** 2026-10-07

**Authors:** Meng Wang, Chihim Mak, Xinjie Li, Shaoyu Li, Chunyan Cui, Defeng Cai, Zhiwei Liang, Tao Lu, Qingshun Jia, Xiangming Shao, Yong Ma, Guanghui Fan, Kejin Li, Wenzhi Mai, Chihang Mak, Nan Liu

**Affiliations:** 1 Institute of Environment and Health, South China Hospital, Medical School, Shenzhen University, Shenzhen, China; 2 Institute of Chronic Disease Risks Assessment, School of Nursing and Health, Henan University, Kaifeng, China; 3 Hong Kong TungTakSim Biotechnology Co., Ltd., Hong Kong, Hong Kong SAR, China; 4 Center of Clinical Laboratory and Pathology, South China Hospital, Medical School, Shenzhen University, Shenzhen, China; 5 School of Pharmacy, Guangzhou University of Chinese Medicine, Guangzhou, China; 6 School of Life Sciences, Beijing University of Chinese Medicine, Beijing, China; 7 Department of Internal Medicine, Shandong Xinzhonglu Traditional Chinese Medicine Hospital, Jinan, China

**Keywords:** amyotrophic lateral sclerosis (ALS), herbal nutritional intervention, *Klebsiella* *pneumoniae* (KP), multidrug-resistant organism (MDRO), respiratory failure

## Abstract

We report a retrospective single case of a female patient with a 9-year history of amyotrophic lateral sclerosis (ALS) who presented with respiratory failure due to a multidrug-resistant organism (MDRO) infection, predominantly *Klebsiella pneumoniae* (KP), and who showed significant clinical improvement during a comprehensive treatment regimen that included an adjunctive herbal nutritional intervention. The objective of this retrospective single-case report was to describe the clinical course of this patient following a nutritional intervention with two herbal beverages: Sang Shen Pu Gong Ying (SSPGY) and Wu Zhi Mao Tao Hong Zao (WZMTHZ). The patient was admitted in critical condition and managed in three phases: initial antibiotics with occasional herbal beverages, daily herbal beverages, and a combination of herbal beverages with intermittent antibiotics. Retrospective analysis of clinical data revealed significant improvements post-intervention. Body weight increased from 26 kg to 35.5 kg at discharge and 39 kg at the 6-week follow-up. Vital signs normalized, with no detectable KP in the final hospital phase. Inflammatory markers, including C-reactive protein and white blood cell count, decreased markedly. Neurological status, as indicated by neurofilament light chain (NfL) levels, improved, and the patient showed respiratory autonomy and positional changes. Nutritional markers, total protein and creatinine, also improved. This case documents clinical improvements, including extended ventilator-free breathing periods, in an ALS patient with MDRO infection following a combined therapeutic approach. Although concurrent medical interventions were administered in real-world clinical practice, these findings suggest the potential value of this adjunctive intervention, warranting further systematic investigation.

**Clinical Trial Registration**: identifier ITMCTR2024000322.

## 1 Introduction

The emergence of antimicrobial resistance (AMR) anywhere can quickly become a global problem (1). The World Health Organization (WHO) has issued a comprehensive report highlighting the global spread of a particularly concerning pathogen, *Klebsiella pneumoniae* (KP) (https://www.who.int/emergencies/disease-outbreak-news/item/2024-DON527). The dissemination of KP is a matter of grave concern due to its potential to cause widespread health issues. KP is a prevalent member of the multidrug-resistant organism (MDRO) category, which is a critical challenge in contemporary medicine (2). KP is a common MDRO. In clinical settings, it accounts for approximately one-third of hospital-acquired pneumonia cases, while approximately half of pneumonia-related deaths are associated with multidrug resistance (3). According to current estimates, bacterial infections claim 7.7 million lives annually, with drug-resistant pathogens being linked to 4.95 million of these deaths (4). Therefore, there is an urgent need for the development of innovative therapeutic strategies to counteract the escalating threat of multidrug-resistant infections (5). In severe cases, KP infection can progress to respiratory failure or circulatory collapse. Additionally, there is a risk of complications such as lung abscesses, empyema, pericarditis, or meningitis, which underscores the gravity of KP infections and the need for vigilant clinical management (6).

Amyotrophic lateral sclerosis (ALS) is a devastating neurodegenerative disorder characterized by the progressive degeneration of both upper and lower motor neurons. The underlying causes and mechanisms of ALS remain elusive, further contributing to the complexity of the disease. ALS not only poses a significant risk for pulmonary infections due to the compromised respiratory musculature but also necessitates aggressive medical interventions (7). Unfortunately, the trajectory of ALS is marked by extended hospital stays, substantial financial burdens, and high in-hospital mortality rates, with few patients achieving routine discharge. These factors highlight the critical need for innovative therapeutic approaches and supportive care strategies for individuals afflicted with ALS.

At present, there are no effective treatment options for critically ill patients with respiratory failure complicated by ALS and MDRO infection. In this challenging clinical setting, recent research has highlighted the potential of herbal-based nutritional interventions in the management of these severe infectious diseases. For example, studies have demonstrated that specific nutritional formulations can significantly modulate immune responses and improve clinical outcomes in infection management (8), and the concept of “medicine-food homology” (a traditional theory asserting that certain dietary ingredients offer pharmacological benefits alongside their nutritional value) has gained increasing attention as an approach for providing both essential nutritional support and therapeutic benefits in critical care. Here, we present what may be the first retrospective single-case report of a critically ill ALS patient with a multidrug-resistant infection who received an adjunctive nutritional approach involving two herbal beverages: Sang Shen Pu Gong Ying (SSPGY) and Wu Zhi Mao Tao Hong Zao (WZMTHZ). Both SSPGY and WZMTHZ beverages are commercial products manufactured and standardized under strict quality control protocols by Hong Kong TungTakSim Biotechnology Co., Ltd. Due to intellectual property protections, the exact extraction parameters and precise ingredient ratios remain proprietary. To ensure clinical reproducibility, the beverages were continuously stored at room temperature and protected from light. During the intervention, the formulations were administered directly in their original undiluted form via a nasogastric tube, without further dilution or preparation. The core active ingredients of SSPGY include *Morus alba* fruit, *Taraxacum mongolicum*, *Zingiber officinale*, and *Momordica charantia*, while WZMTHZ primarily consists of *Ficus hirta*, *Ziziphus jujuba*, *Morus alba* fruit, and *Dioscorea opposita*. The complete botanical compositions of both beverages are detailed in Supplementary Table S1. Following this combined interventional course, vital signs and various inflammatory indicators gradually returned to normal levels, and microbiological clearance of KP was ultimately achieved, as evidenced by subsequent negative cultures. At the 6-week follow-up post-discharge, the patient was able to be ventilator-free for 3–5 h per day and maintained the ability to rest comfortably in a supine position.

## 2 Case report

A 54-year-old woman with a 9-year history of amyotrophic lateral sclerosis (ALS), a 4-year history of uncontrolled hypertension, and extreme malnutrition was admitted to a tertiary care hospital (Hospital A) from December 13 to 30, 2023. Her initial symptoms included a 4-day fever of unclear etiology with a peak temperature of 38° C, a cough, and shortness of breath. Her respiratory symptoms were exacerbated when supine, but she experienced some relief upon sitting. There were no complaints of chest pain, nasal congestion, or urinary symptoms. Initial treatment at a community clinic with oral amoxicillin clavulanate potassium and loratadine resolved the fever temporarily, but respiratory symptoms persisted, and her oxygen saturation dropped to approximately 80%. 1 h prior to hospital presentation, she experienced a sudden episode of severe dyspnea and loss of consciousness without convulsive movements or incontinence. Upon arrival at the emergency department, she was intubated and placed on invasive ventilation, and an intravenous line was established. Blood gas analysis revealed a respiratory acidosis with compensatory metabolic alkalosis (pH: 7.12, PCO_2_: 89.2 mmHg, PO_2_: 118 mmHg, Na^+^: 139 mmol/L, K^+^: 3.6 mmol/L, Lac: 2.54 mmol/L, HCO_3_^−^: 27.8 mmol/L, BE: −1.5 mmol/L). A chest CT scan revealed an infection in the right lower lobe, and she was admitted to the ICU with a diagnosis of respiratory failure.

In the ICU, she received antimicrobial therapy, inotropic support, mucolytics, bronchodilators, therapies to support cardiac function, and electrolyte management, alongside a tracheotomy to maintain airway patency. Concurrently, adjunctive herbal nutritional interventions were administered: on day 2 after admission, oral WZMTHZ was initiated at a dosage of 600 mL per day for 4 days. Subsequently, on day 4 after admission, oral administration of SSPGY was started at 50–100 mL per dose, three times daily, for a duration of 14 days.

A sputum culture 10 days later revealed that KP was susceptible only to tigecycline. A follow-up chest CT on day 16 of admission revealed worsening pulmonary infection, with worsening fever and increased respiratory secretions (Figure 1A). Given the lack of therapeutic response, her family requested discharge and transfer to a tertiary hospital (Hospital B) for further treatment.

**Figure 1:**
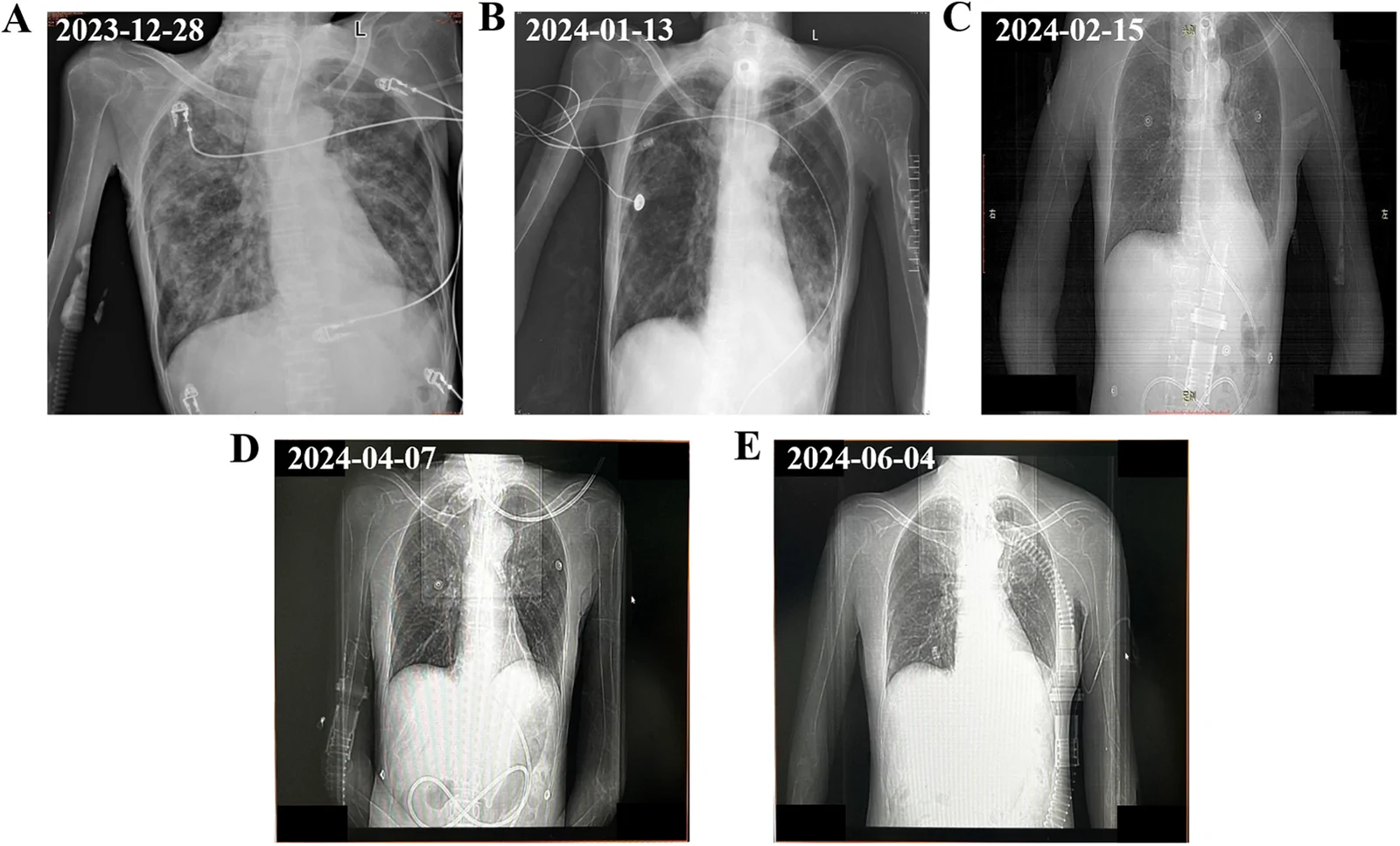
CT imaging changes in both lungs. **(A)** Signs of infection in both lungs on December 28, 2023; **(B)** signs of infection and a left-sided pleural effusion on January 13, 2024; **(C)** multiple areas of bilateral pulmonary inflammation, partial resolution of the pulmonary inflammation, and left pleural effusion with poor expansion of the left lower lobe of the lung on February 15, 2024; **(D)** near-complete resolution of bilateral pulmonary inflammation and pleural effusion was observed on April 7, 2024; **(E)** signs of bilateral pulmonary inflammation at the 6-week follow-up.

From December 30, 2023, to January 14, 2024, the patient's condition deteriorated and she was admitted to the ICU at a tertiary hospital with her body weight (BW) dropping to 26 kg (Supplementary Figure S1). She underwent daily bedside bronchoscopy with bronchoalveolar lavage for 14 consecutive days, which revealed abundant yellowish secretions, while microbiological cultures consistently confirmed KP infection. Antimicrobial therapy with tigecycline and polymyxin B was initiated but yielded no significant improvement, and *Pseudomonas aeruginosa* was detected in her urine. On day 13, she developed septic shock with escalating inflammatory markers (Figure 1B), prompting a switch to ceftazidime-avibactam. Given the lack of clinical response to the treatment and the patient's deteriorating condition, the family opted to transfer her to another hospital (Supplementary Video S1). Her family members reported that, due to the deterioration of her condition, her daily intake of SSPGY beverages was reduced to less than 150 mL, and sometimes none at all.

From January 17 to March 1, 2024, she was transferred to a rehabilitation hospital, where a Critical Illness Notification was issued upon admission (Supplementary Figure S1). Cultures confirmed persistent KP infection, and tigecycline treatment was discontinued on February 8 after no clinical improvement. During this period, the patient initially received SSPGY beverages via a nasogastric tube at a dosage of 600–900 mL per day for approximately 20 days. A repeat sputum culture showed no KP growth after two days without antibiotics. Subsequently, the intervention was switched to WZMTHZ beverages at a dosage of 300 mL per day for about 20 days, during which a follow-up CT scan indicated partial resolution of the pulmonary inflammation (Figure 1C). Although new infections with *P. aeruginosa* and *Escherichia coli* were detected in her urine, her inflammatory markers and level of consciousness improved. By March 1, she was transferred back to a local tertiary hospital for routine care, where she remained from March 1 to April 20, 2024. During this period, the patient's condition stabilized, with intermittent anti-infective therapy and standard supportive care. Concurrently, family members continued to administer 600 mL of SSPGY beverages daily for approximately 20 days (Supplementary Videos S2 and S3). Upon completion of this course, the intervention was switched to WZMTHZ beverages, with the dosage gradually increasing from 300 to 600 mL per day, which was maintained continuously through the discharge and follow-up period. Intermittently during this course, a solution of 40 mL of SSPGY diluted in 1,000 mL of normal saline was utilized for bladder irrigation (Supplementary Videos S4 and S5). On March 23, sputum culture showed no pathogenic growth. However, the same sputum sample was sent to South China Hospital of Shenzhen University on March 29 and tested positive for *P. aeruginosa*. Subsequent sputum samples, collected simultaneously at both Hospital A and South China Hospital of Shenzhen University one week later, showed no KP growth. On April 7, a chest CT scan demonstrated significant resolution of lung inflammation and pleural effusion (Figure 1D). Throughout her stay, three sputum cultures were consistently positive for *P. aeruginosa*. The patient was deemed clinically stable and was discharged for home nursing on April 20, 2024. To ensure comprehensive reporting of the clinical course, the detailed intervention timelines—including observation periods, herbal beverage regimens (type, volume, and nasogastric administration), concurrent antibiotic therapies, in-hospital nutritional and symptomatic supportive care, and corresponding microbiological culture outcomes—are systematically summarized in the Supplementary Flowcharts.

At a 6-week post-discharge follow-up, infection indicators (including WBC, CRP, and PCT) had normalized and sputum analysis showed no pathogenic growth. However, a urine culture still detected *P. aeruginosa*, likely due to prolonged urethral catheterization. Despite persistent bilateral lung inflammation as observed on CT (Figure 1E), the patient's overall clinical status improved significantly. She remained conscious for most of the day, engaged in passive limb exercises, and tolerated extended ventilator-free periods (3–5 h daily).

## 3 Results

### 3.1 Recovery of vital signs and physiological function

We conducted a longitudinal evaluation of the patient's health status at baseline (pre-study), followed by assessments at 2, 8, and 16 weeks during the administration of the herbal beverages, and finally at a 6-week post-discharge follow-up. The indices of vital signs, inflammation, and neurodegenerative disease-related parameters during hospitalization and follow-up are presented in Table 1.

**Table 1:** Vital signs, inflammation markers and neurodegenerative disease-related parameters during hospitalization and follow-up.

Variable	Prestudy	2w	8w	16w	6w Follow-up	Normal value or range
Weight (kg)	–	26	30	35.5	39	–
Body-mass index	–	10.16	11.72	13.87	15.23	18.5–23.9
Blood pressure (mmHg)	100/52	115/67	127/59	126/85	125/86	90–140
Heart rate (beats/min)	101	110	81	98	81	60–100
Respiratory rate (breaths/min)	18	15	23	21	20	16–20
Blood index						
Absolute neutrophil count (10^9^/L)	7.60	11.36	3.33	5.90	5.0	1.8–6.3
Absolute lymphocyte count (10^9^/L)	6.20	0.76	1.45	0.90	2.1	1.1–3.2
WBC (10^9^/L)	14.48	12.5	5.12	7.32	7.60	3.5–9.5
CRP (mg/L)	–	149	3.80	5.60	1.30	0–5
PCT (ng/mL)	–	0.29	0.10	0.11	0.16	0.01–0.5
NfL (pg/mL)	–	–	2,615.65	–	368.63	0.00–30.50

The results revealed a progressive increase in both BW and body mass index (BMI). In particular, BW increased from a nadir of 26 kg (recorded on December 30, 2023) to 39 kg (recorded on June 17, 2024) at follow-up, which was accompanied by an amelioration of severe malnutrition and a concomitant improvement in metabolic and multi-organ functions. Upon hospitalization, the patient presented with shock-induced hypotension, as evidenced by a BP of 64/40 mmHg (Supplementary Figure S3) on February 17, 2024. During treatment with vasopressors, marked fluctuations in BP were observed, peaking at 154/105 mmHg (Supplementary Figure S4) on the same day, which posed a considerable risk for a critically ill individual. Following the initiation of the adjunctive herbal nutritional intervention, alongside the titration of vasoactive medications, the patient's BP gradually stabilized within a normal range. Concurrently, vital sign indicators such as heart rate and respiratory rate also stabilized, suggesting that the patient's overall physiological status had progressively stabilized.

### 3.2 Analysis of antibacterial effects

In this clinical case, the patient received antibiotic therapy during the first 8 weeks of hospitalization, the initial antimicrobial regimens included biapenem (0.3 g/day, intravenous infusion, for 3 days), levofloxacin (0.2 g/day, intravenous infusion, for 2 days), cefoperazone–sulbactam (1.5 g every 8 h, intravenous infusion, for 7 days), and cefotaxime (2 g every 8 h, intravenous infusion, for 7 days). With disease progression, the antimicrobial regimen was escalated to include meropenem (1 g every 8 h, administered via infusion pump, for 4 days), tigecycline (50 mg every 12 h, administered via infusion pump, for 12 days), polymyxin B (250,000 IU and 375,000 IU alternately every 12 h via intravenous infusion pump, for 10 days), and ceftazidime–avibactam (2.5 g every 8 h via intravenous infusion pump, for 2 days).

From December 17, 2023, to January 14, 2024, the patient received low-dose SSPGY decoction in divided doses through a nasogastric tube according to clinical tolerance. For descriptive purposes, daily SSPGY administration of less than 600 mL was categorized as low-dose exposure in this case. During this period, KP remained detectable in sputum cultures. From January 25 to February 8, 2024, the patient received SSPGY at a dose of 600 mL/day. This phase was not exclusively attributable to SSPGY intervention, as tigecycline (50 mg administered intravenously every 12 h) was concurrently administered from January 28 to February 8 for a total duration of 12 days. A follow-up sputum culture obtained on February 10 was negative for KP.

From February 9 to February 29, 2024, the patient received no antibiotics. However, KP was again detected in sputum culture on February 27. SSPGY administration at 600 mL/day was resumed from March 1 and continued until March 19. During the same hospitalization, the patient also received routine inpatient treatments, including intravenous cefoxitin sodium (2 g every 12 h for 2 days), gentamicin bladder irrigation (240 mg/day for 11 days), and subsequent gentamicin bladder irrigation (160 mg/day for 13 days). Additionally, the patient received single-dose bladder irrigations with gentamicin sulfate on March 2, March 7, and April 4, 2024, at doses of 240 mg, 160 mg, and 240 mg, respectively. Notably, antimicrobial susceptibility testing of the KP isolate obtained on February 27 demonstrated resistance to both cefoxitin and gentamicin. Therefore, these antimicrobial agents were administered as part of routine inpatient management rather than as susceptibility-guided targeted therapy against the KP isolate. No subsequent cultures yielded KP during weeks 12–18 and the 6-week post-discharge follow-up. The detailed Antimicrobial Susceptibility Testing (AST) results across various clinical time points and specimen types are summarized in Supplementary Table S2.

Over the course of treatment, the patient's inflammatory markers progressively normalized, as detailed in Table 1. During the observation period, the patient received intermittent antibiotic therapy, with the specific agents, dosages, administration frequencies, and treatment periods described above and summarized in the Supplementary Flowcharts. These biomarker changes were consistent with an improvement in the patient's overall clinical status during the comprehensive treatment period, which included the adjunctive administration of SSPGY beverages, potentially supporting her rehabilitation efforts. Importantly, the patient experienced no adverse effects during the consumption of these beverages.

### 3.3 Treatment outcomes in a patient with comorbidities

This patient, who had an MDRO infection, also had a 9-year history of ALS, which had rendered her limbs non-functional. During the 8-week observation period, the patient received comprehensive management, including an herbal-based nutritional intervention and supportive care as described in Section 3.2 and the Supplementary Flowcharts. After this period and a subsequent 6-week follow-up, the patient's neurofilament light (NfL) levels decreased markedly, from 2615.65 pg/mL to 368.63 pg/mL. The post-discharge follow-up revealed that the patient could breathe without ventilatory support for 3-5 h per day, as documented in Supplementary Videos S6–S11. Furthermore, the patient's positioning shifted from the long-standing semi-seated or lateral posture to a supine position, as detailed in Supplementary Videos S12 and S13. Observations of the patient's fingers and toes in the follow-up video indicated increased mobility. These observations reflect substantial improvements in the patient's respiratory capacity, physical activity, as well as in aspects of her daily functioning and quality of life.

### 3.4 Safety and nutritional status assessments of the herbal-based beverages for the patient

A comprehensive analysis of the hepatorenal function data from the patient was conducted. Following the consumption of SSPGY beverages, the liver function indices including alanine aminotransferase (ALT), aspartate aminotransferase (AST), alkaline phosphatase (ALP), gamma-glutamyltransferase (GGT), direct bilirubin (DBIL)/indirect bilirubin (IBIL)/total bilirubin (TBIL), total bile acid (TBA), total protein (TP), albumin (ALB), and globulin (GLO) remained stable and within the normal ranges, indicating stable hepatic function (Figures 2A–C). Despite considerable fluctuations in TP/ALB/GLO levels, as illustrated in Figure 2D, these values still demonstrated an overall improving trend. Creatinine (CRE) and uric acid (UA) are important parameters in evaluating renal function. UA levels fluctuated during the early phase but returned to the normal range from day 56 after intervention onward; CRE levels remained within the normal range from day 45 after intervention onward. As shown in Figure 2F, the patient's blood Urea nitrogen (BUN) and Urea levels showed mild fluctuations, while BUN levels remained generally stable throughout the follow-up period. Although Urea levels fluctuated during the intervention period, they generally remained within the normal range from day 28 onward. From the end of treatment through discharge and follow-up, Urea levels showed a gradual upward trend, indicating that the patient's nutritional status was gradually recovering.

**Figure 2:**
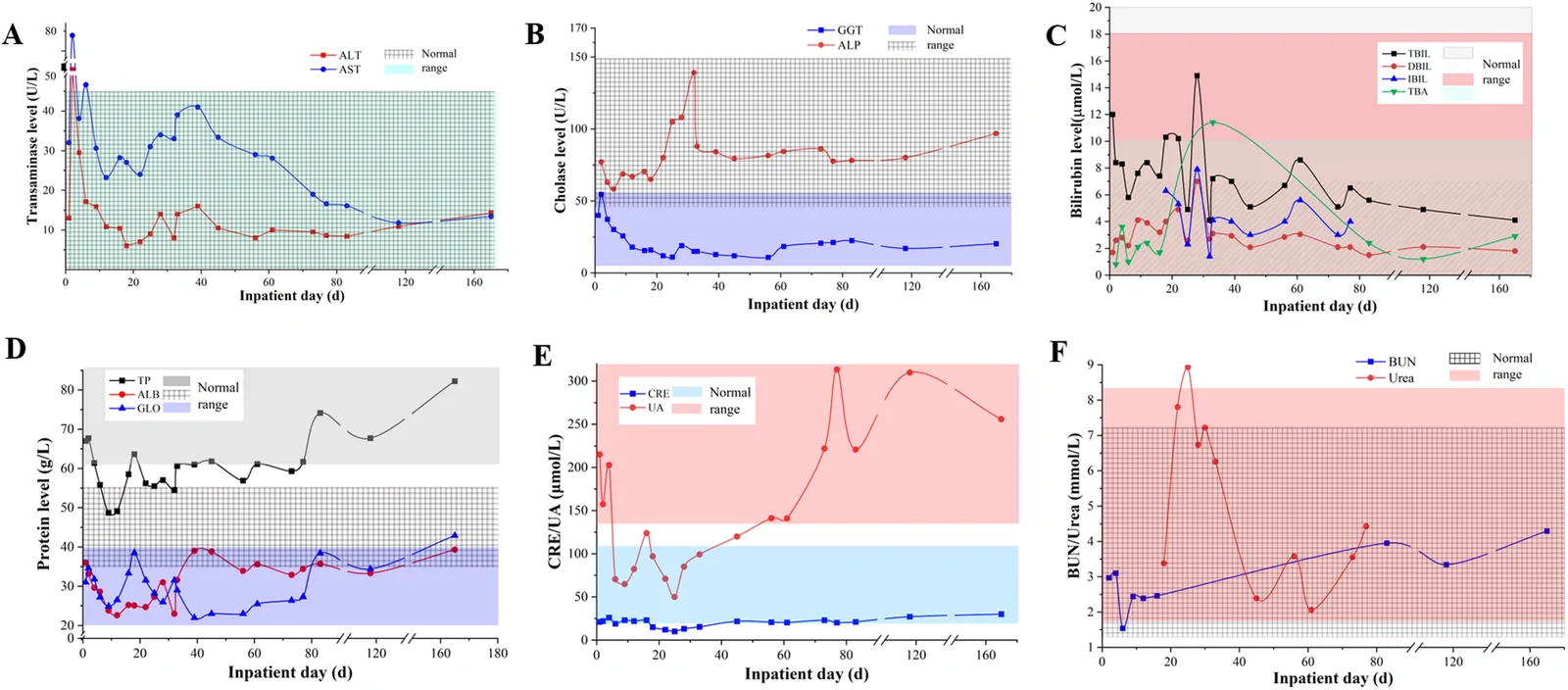
Trends of liver and renal function changes in the patient during treatment. **(A)** ALT and AST; **(B)** ALP and GGT; **(C)** DBIL, IBIL, TBIL and TBA; **(D)** TP, ALB and GLO; **(E)** CRE and UA; **(F)** BUN and Urea.

These findings suggest that the herbal-based beverages were well-tolerated by the patient, with no significant adverse effects on hepatic or renal function. However, the potential risk of gastrointestinal intolerance in critically ill ALS patients with impaired swallowing function cannot be completely ruled out.

## 4 Discussion

This case highlights the challenges of managing severe infections and nutritional deficits in a patient with advanced ALS, demonstrating the complexities of antimicrobial resistance, critical care, and rehabilitation. Herbal remedies have been a cornerstone in the treatment of infectious diseases for centuries, and recent literature suggests they possess potential adjunctive value in combating drug-resistant pathogens. A variety of bioactive compounds have been reported to exhibit antimicrobial properties (9, 10). As introduced earlier, SSPGY and WZMTHZ are medicinal food beverages that have been explored for nutritional support in seriously ill patients, including those infected with COVID-19 (11, 12).

The clinical rationale for selecting the SSPGY and WZMTHZ formulations was based on a dual-targeted therapeutic strategy, informed by both traditional “medicine-food homology” theories and prior empirical studies. The patient presented with a highly complex pathology: a severe MDRO infection requiring aggressive microbial clearance, compounded by end-stage ALS and extreme malnutrition requiring urgent physiological and metabolic restoration. According to traditional formulation principles, SSPGY is designed for “clearing heat and detoxifying” (Qingre Jiedu). Its key ingredients possess well-documented anti-inflammatory and broad-spectrum antimicrobial properties, specifically targeting the pulmonary infection and systemic inflammatory storm. Conversely, WZMTHZ focuses on “strengthening body resistance and consolidating the constitution” (Fuzheng Guben). Its ingredients are recognized for their multi-target immunomodulatory effects and ability to enhance gastrointestinal nutrient absorption. This provided the essential nutritional substrate to help ameliorate the patient's critical cachexia and support immune reconstitution. In this case report, we present a critically ill patient with ALS who experienced multiple organ failure due to a severe MDRO infection. She faced numerous transfers into several ICUs and had a period during which treatment was discontinued (Supplementary Figure S2). Following the combined medical and herbal nutritional intervention, specifically SSPGY and WZMTHZ, there was a notable stabilization in the patient's vital signs, particularly BP. This observation suggests that the long-term maintenance of BP in critically ill patients might not be sustainable with vasoactive drugs alone, indicating a need for a more comprehensive approach to restoring physiological homeostasis. This suggests a potential adjunctive role for herbal supplements in nutritional and physiological regulation. Furthermore, the clinical symptoms of infection, including fever, cough, and fatigue, showed significant improvement during this comprehensive treatment period.

These observed clinical improvements may be partially attributed to the pharmacological properties of key ingredients such as mulberry and dandelion. Mulberry is enriched with polyphenols and vitamins, which contribute to its antioxidant and immunomodulatory effects that may enhance the immune response, modulate inflammation, and inhibit the proliferation of pathogenic microorganisms (13, 14). Dandelion has anti-inflammatory and diuretic properties and is traditionally regarded as having detoxifying effects. Studies have shown that polysaccharides from dandelion root extract exhibit immunomodulatory activity (15). Existing pharmacological literature suggests that several key botanical ingredients within the beverages possess inherent antimicrobial properties. We hypothesize that the observed clinical clearance of KP may be partially associated with the combined antibacterial activities of these components. For instance, *Taraxacum* species (dandelion) are rich in phenolic acids and flavonoids, which have been documented to exhibit broad-spectrum antibacterial activity, acting as a potential source of antibacterial agents against various pathogens (16). Additionally, active compounds in *Zingiber officinale* (ginger) have demonstrated significant antibacterial activity, exhibiting strong inhibitory effects specifically against multidrug-resistant clinical pathogens, including KP (17).

The patient had a 9-year history of ALS, with severely compromised immune function, predisposing her to infections. In this case, the infection led to a deterioration in the patient's health, culminating in multiple hospital transfers, and even the issuance of a Critical Illness Notification by the hospital. The patient was 160 cm tall, had a BW as low as 26 kg, translating to a BMI of approximately 10.16, indicative of severe malnutrition. The patient's nutritional and metabolic status improved significantly. The patient's body weight increased significantly from 26 kg to 39 kg (Table 1), accompanied by improvements in levels of TP and CRE. This suggests enhanced nutritional status and metabolic recovery, potentially due to the comprehensive care, including the nutrient-dense composition of the beverages. The blend of botanical ingredients may have improved gut health, nutrient absorption, and systemic metabolism, contributing to the recovery.

She exhibited extreme emaciation, with a profound depletion of muscle and fat reserves, which heightened the risk of multiple organ failure, infectious complications, and a significantly elevated mortality risk. Under these circumstances, incorporating adjunctive herbal nutritional support into the intervention strategy emerged as a critical therapeutic option. The marked changes in the patient's BW and clinical status under herbal nutrition support signify a notable amelioration of extreme malnutrition and an improvement in the patient's multiple organ failure, thereby reducing the risk of mortality and improving the prospects for recovery.

The patient's inflammatory markers, including absolute neutrophil and lymphocyte counts, WBC, CRP and PCT (ng/mL) (Table 1), showed marked decreases during the comprehensive intervention. This suggests that the herbal beverages may exert anti-inflammatory effects, potentially mediated by the bioactive compounds including polyphenols (18) and polysaccharides (19) in mulberry and dandelion.

Serial blood tests for liver and kidney function during the administration of the two herbal nutritional beverages revealed that her ALT, TBIL, BUN and Urea levels were maintained within the normal range, suggesting that the herbal nutritional beverages were well-tolerated in this case, without adversely affecting hepatic and renal functions. During the follow-up, we observed a gradual weaning from ventilator dependence, with the patient achieving independent breathing for 3–5 h per day, which represented a marked improvement from the initial reliance on mechanical ventilation. This indicated a significant recovery in physiological function. A significant decrease in NfL levels from 2615.65 pg/mL to 368.63 pg/mL was observed. NfL is considered one of the biomarkers of ALS, and this decrease aligns with the observed improvements in motor function (20) and respiratory autonomy (21). The patient's motor functions showed slight improvements, with the ability to move limbs and a change in positioning from a prolonged semi-seated or lateral posture to a supine position. However, as this is a single retrospective case involving multiple interventions, the reduction in NfL levels and partial motor-function improvement may be secondary to the successful control of the infection and the overall improvement in systemic nutritional status.

In real-world clinical practice, hospitalized patients must inherently receive standard-of-care treatments according to prescribed medical protocols, rendering it impossible to completely eliminate clinical confounding factors. However, by longitudinally comparing the patient's clinical status before and after the introduction of the herbal beverages, it is clinically reasonable to hypothesize that the nutritional support provided by these botanical interventions is positively associated with her favorable clinical trajectory. Nevertheless, considering that this remains a single-case observation, these preliminary findings cannot yet be generalized to a broader patient population, and the following specific limitations must be rigorously acknowledged: (1) Single-case and retrospective nature: As this is a retrospective single-case observation, the findings are subject to inherent biases in data collection and interpretation. (2) Confounding medical interventions and lack of a control group: Crucially, the absence of a control group and the presence of multiple concurrent medical interventions (such as intermittent antibiotics, bronchoscopic bronchoalveolar lavage, and standard enteral nutrition) make it impossible to definitively attribute the observed clinical improvements solely to the herbal beverages. (3) Limited generalizability: Because causality cannot be definitively established in this isolated clinical scenario, these preliminary findings cannot be generalized to broader ALS or MDRO patient populations. (4) Call for further research: To validate these preliminary observations and establish clinical efficacy, we emphasize the need for larger-scale, multicenter, randomized controlled trials to confirm the therapeutic potential of SSPGY and WZMTHZ beverages. These studies should focus on understanding the mechanisms of action, long-term efficacy, and safety profiles of these adjunctive interventions.

## 5 Conclusion

In conclusion, this retrospective single-case report describes the clinical course of a patient with ALS complicated by a multidrug-resistant organism infection who received SSPGY and WZMTHZ beverages alongside standard medical care. Despite the patient's extreme immunodeficiency and infection with an MDRO, remarkable clinical improvements were observed during the comprehensive treatment period, which included the herbal-based nutritional intervention. This observation highlights the potential adjunctive value of herbal nutritional support in critical care, suggesting it may serve as a beneficial complement to standard emergency care and treatment for severe infections. However, due to the single-case design and concurrent medical therapies, direct causality cannot be definitively established. Further systematic investigations will focus on the pharmacological properties and safety profiles of the herbal beverages and the application of medicine-food homology-based formulations in anti-infective treatment. This research may provide a promising direction for herbal nutritional therapies in MDRO infections and ALS and may also inform the development of new adjunctive strategies for severe diseases and emergency care.

## Data Availability

The original contributions presented in the study are included in the article/Supplementary Material, further inquiries can be directed to the corresponding authors.
