# Supplementary material for: Case Report: Herbal nutritional intervention in a critically ill ALS patient with multidrug-resistant infection
Source: Front Med (Lausanne). 2026 Oct 7;13:1912856. doi: 10.3389/fmed.2026.1912856 (PMC13683183; doi:10.3389/fmed.2026.1912856)
Supplement: Supplementary file 1 [file Supplementaryfile2.pdf]

Flowchart S1 Timeline of clinical examinations and laboratory test results throughout the herbal nutritional intervention.

Hospital A (12/13/2023-12/30/2023)

Date	Anti-infective drug or beverage	Sputum culture	Image examination	Urinalysis
12/13/2023-12/15/2023	Biapenam			
12/14/2023				Red blood cells: 76.4/μL<br>Epithelial cells: 22.4/μL<br>Small round cells: 22.4/μL
12/15/2023			No abnormalities found in the cardiac septum of the two lungs	
12/15/2023-12/18/2023	Wu Zhi Mao Tao Hong Zao Zhi Wu beverage: 3 times/d			
12/17/2023	Cefoperazone Sodium and Sulbactam Sodium 12/16-12/23		Both lower lung texture increased, blurred	
12/17/2023-12/30/2023	Sang Shen Pu Gong Ying beverage: 3 times/d			
12/19/2023				
12/21/2023			Increased texture in both both sides of the lung	
12/23/2023				Red blood cells: 29.1/μL<br>Epithelial cells: 18.4/μL<br>Small round cells: 17.9/μL
12/24/2023			Two pneumonic changes	
12/25/2023-12/30/2023	Cefotaxime; Levofloxacin; Fluconazole			
12/26/2023		*Klebsiella pnenmoniae*		
12/28/2023			Infection both sides of the lung	
12/29/2023	Biapenam 12/30		Infectious lesions fond on both sides of the lung	

Hospital B (12/30/2023-1/14/2024)

Date	Anti-infective drug	Results of bacterial culture (sputum/urine)	Urinalysis	Image examination
12/30/2023	Cefoperazone Sodium and Sulbactam Sodiu		Urobilogen 1+<br>Carcass 2+<br>White blood cells 2+<br>White blood cells:555/μL<br>Yeast:98/μL<br>Small amount of mucus filament	
12/31/2023	Meropenem 12/31/2023-01/04/2023			
12/31/2023-1/12/2024	Tigecycline			
1/1/2024				Infection on both sides of the lung
1/2/2024		*Klebsiella pneumoniae*		
1/2/2024-1/12/2024	Polymyxin B			
1/4/2024				Infection on both sides of the lung, but slightly less partial than Jan. 1
1/5/2024		*Klebsiella pneumoniae*		
1/7/2024				Infection both sides of the lung but slightly less partial than Jan. 4
1/9/2024		*Klebsiella pneumoniae*		
1/10/2024		Two times pulmonary Alveolar lavage		Infection on both sides of the lung found similar to Jan. 7
1/12/2024-1/14/2024	Ceftazidime-avibactam			
1/13/2024				Infection on both sides of the lung with left pleural effusion
1/14/2024		*Pseudomonas aeruginosa*		
1/15/2024		*Klebsiella pneumoniae*		

Hospital C(1/14/2024-1/17/2024)

Date	Sputum culture	Image examination	Urinalysis
1/14/2024	*Baumanii*		
1/15/2024		Left pleural effusion	White blood cell count:78/μL<br>Red cell count:249/μL<br>Epithelial cell count:17/μL<br>White blood cells (high power field):14/H<br>Erythrocyte (high power field):45/H

Hospital D(1/17/2024-3/1/2024)

Date	Anti-infective drug	Alveolar wash	Results of bacterial culture (sputum/urine)	Urinalysis	Image examination
1/18/2024				White blood cellS 2+	
1/19/2024			*Klebsiella pneumoniae*		Multiple inflammations of both lungs
1/23/2024		Pulmonary alveolar lavage			
1/26/2024				White blood cells 3+<br>Occult Blood +<br>White blood cells (microscopy)2+	
1/27/2024-2/8/2024	Tigecycline				
1/29/2024			*Klebsiella pneumoniae*		Multiple inflammations of both lungs
2/10/2024		Pulmonary alveolar lavage	Normal flora growth		
2/11/2024			*Pseudomonas aeruginosa*		
2/15/2024				White blood cells +<br>Vitamin C3+	Multiple inflammation on both lungs along with partial absorption inflammation
2/17/2024		Pulmonary alveolar lavage			
2/19/2024			*Escherichia coli, Stenotrophomonas maltophilia*		
2/20/2024				White blood cells 3+<br>Nitrite +<br>White blood cells (microscopy)2+<br>Vitamin C+	
2/21/2024			*Pseudomonas aeruginosa*		
2/24/2024			*Baumanii*		
2/27/2024			*Klebsiella pneumoniae*	White blood cells 3+<br>Nitrite +<br>White blood cells (microscopy)2+<br>Vitamin C+	The results found similar to Feb. 15 results
2/29/2024		Pulmonary alveolar lavage			

Hospital A(3/1/2024-4/20/2024)

Date	Anti-infective drug	Sputum culture	Image examination	Urinalysis
3/1/2024-3/2/2024	Cefixime sodium<br>Gentamicin sulfate injection 3/2/2024			
3/4/2024-3/29/2024	Gentamicin sulfate injection			
3/6/2024				White blood cells:363.8/μL<br>Red blood cells:87.1/μL<br>Bacteria:8937.4/μL
	Gentamicin sulfate injection 3/7/2024			
3/23/2024		No growth of pathogenic bacteria		
4/3/2024		White blood cells< 25/LP<br>Epithelial cells < 10/L		
	Gentamicin sulfate injection 4/4/2024			
4/5/2024		*Pseudomonas aeruginosa*		
4/7/2024			Double pneumonic lesions with pleural effusion but basically absorbed than Dec. 29, 2023	
4/8/2024		*Pseudomonas aeruginosa*		
4/13/2024-4/14/2024	Levofloxacin; Cefoperazone Sodium and Sulbactam Sodium			
4/13/2024		*Pseudomonas aeruginosa*	Inflamatory leisions in both lungs with multiple fibrious streaks	

Flowchart S2 Initial clinical diagnoses and identified comorbidities upon hospital admission

Date	Admission diagnosis
Hospital A (12/13/2023-12/30/2023)	1. Coma<br>2. Type II respiratory failure<br>3. Acute coronary syndrome<br>4. Amyotrophic lateral sclerosis (ALS)<br>5. Respiratory acidosis<br>6. Hyperlacticemia<br>7. Pneumonia
Hospital B (12/30/2023-1/14/2024)	1. Severe pneumonia<br>2. Successful resuscitation after Cardiac arrest<br>3. Respiratory failure<br>4. Pneumonia klebsiella bacillus infection<br>5. Bilateral pleural effusion<br>6. Electrolyte disorder: hyperkalemia, hyponatremia<br>7. Amyotrophic lateral sclerosis (ALS)<br>8. High blood pressure level 1<br>9. Fractures of postoperative femoral head
Hospital C (1/14/2024-1/17/2024)	1.Respiratory failure<br>2.Severe pneumonia<br>3.Successful resuscitation after Cardiac arrest<br>4.Producing ultra broad spectrum beta lactamase pneumonia klebsiella bacteria (ESBLS)<br>5.Pleural effusion<br>6.Cardiac insufficiency<br>7.Amyotrophic lateral sclerosis (ALS)<br>8.Iron deficiency anemia<br>9.Hypoalbuminemia<br>10.Coagulant function abnormality<br>11.The electrolyte disorder<br>12.Sepsis<br>13.Compressive zone pressure ulcers<br>14.Asthmatic syndrome (phlegm stasis syndrome)
Hospital D (1/17/2024-3/1/2024)	1. Amyotrophic lateral sclerosis (ALS)<br>2. Anemia (moderate)<br>3. Respiratory failure<br>4. Pneumonia<br>5. Irrhythmia
Hospital A (3/1/2024-4/20/2024)	1. Lung infection<br>2. Chronic respiratory failure (type I respiratory failure)<br>3.Chronic heart failure<br>4. Amyotrophic lateral sclerosis (ALS)<br>5. Pleural effusion<br>6. lacunar infarction<br>7. Hypoalbuminemia<br>8. Malnutrition

Flowchart S3 Detailed administration protocol and chronological process of the nutritional intervention (SSPGY and WZMTHZ).

Note: EN =enteral nutrition; PN(AA) = parenteral nutrition: amino acids; ALB = albumin

Hospital	Date	WZMTHZ	SSPGY	In-hospital nutritional support
Hospital A	12/15/2023-12/18/2023	600 mL/d	Unused	EN + PN(AA) + ALB
	12/17/2023		50 mL/time, 3 times/d	
	12/18/2023-12/30/2023		100 mL/time, 3 times/d	EN(enhanced) + PN(AA) + ALB
Hospital B	1/4/2024-1/14/2024		Less than 150 mL/d	EN + ALB(repeated)
Hospital C	1/14/2024-1/17/2024		600 mL/d	EN 500ml/d + PN(AA-VB) 400ml/d + ALB 10g
Hospital D	1/17/2024-1/24/2024		900 mL/d	EN(SP) 1000ml/d + PN(AA-III) + Fat emulsion 250ml/d + ALB 5g
	1/25/2024-2/8/2024		600 mL/d	EN(SP) 500ml/d + PN(AA-III)
	2/10/2024-3/1/2024	300 mL/d	Quit drinking	EN(SP) 500ml/d(continued)
Hospital A	3/1/2024-3/19/2024		600 mL/d	EN + PN(AA-SF) 21.2g/d
	3/20/2024	300-600 mL/d	Quit drinking	EN (continued until discharge)
	3/20/2024-6/11/2024	600 mL/d	Quit drinking	
	3/30/2024		Bladder irrigation	
