# Supplementary material for: Case Report: Herbal nutritional intervention in a critically ill ALS patient with multidrug-resistant infection
Source: Front Med (Lausanne). 2026 Oct 7;13:1912856. doi: 10.3389/fmed.2026.1912856 (PMC13683183; doi:10.3389/fmed.2026.1912856)
Supplement: Supplementary file 2 [file Supplementaryfile1.docx]

**Figure S1**

**Figure S1** The body weight (26 kg) of patient in the resident admit note.

**Figure S2**

**Figure S2** A critical illness Notification issured by the hopital on Jan. 17, 2024.

**Figure S3**

**Figure S3**  A shock hypotension by instantaneous blood pressure of 64/40 mmHg in the presented intensive rehabilitation nursing record sheet on Feb. 17, 2024

**Figure S4**

**Figure S4** A instantaneous and sudden rising of blood pressure of 154/105 mmHg in the presented intensive rehabilitation nursing record sheet on Feb. 17, 2024.
