# Supplementary material for: Case Report: Herbal nutritional intervention in a critically ill ALS patient with multidrug-resistant infection
Source: Front Med (Lausanne). 2026 Oct 7;13:1912856. doi: 10.3389/fmed.2026.1912856 (PMC13683183; doi:10.3389/fmed.2026.1912856)
Supplement: Supplementary file 3 [file Supplementaryfile3.docx]

**Supplementary Videos**

The supplementary video files associated with this article are available in the Figshare repository. Due to file size and format limitations of the submission system, the high-resolution video materials have been hosted externally to ensure accessibility.

Dataset Title: **Herbal nutritional intervention in a critically ALS patient with multidrug-resistant infection**

Access Link (DOI): The videos can be viewed and downloaded at the following persistent link: [**https://doi.org/10.6084/m9.figshare.31134400**](https://doi.org/10.6084/m9.figshare.31134400)

Video Descriptions:

- **Video S1:** Baseline Condition and Medical History (2024-01-13). Narration by the patient's son documenting the critical condition of the patient and the difficult decision-making process regarding treatment options prior to the intervention.
- **Video S2:** Administration of Herbal Nutritional Intervention (2024-03-21). Demonstration of the daily administration process of the herbal beverage (SSPGY). The patient is shown receiving the first bottle of the nutritional support.
- **Video S3:** Administration of Herbal Nutritional Intervention (2024-03-21). Continued documentation of the administration of the SSPGY herbal beverage (second bottle/dosage), highlighting the patient's compliance and swallowing ability.
- **Video S4:** Routine Nursing Care: Bladder Irrigation (2024-03-30). Video recording of the bladder irrigation procedure performed on the patient to prevent urinary tract infection and maintain catheter patency.
- **Video S5:** Routine Nursing Care: Bladder Irrigation (2024-03-31). Follow-up documentation of the bladder irrigation procedure on the subsequent day, showing consistent nursing care management.
- **Video S6:** Ventilator Weaning Process - Session 1 (2024-07-10, 15:49). Documentation of the patient's respiratory function improvement. The video shows the patient successfully weaning off the ventilator and breathing spontaneously in the afternoon.
- **Video S7:** Ventilator Weaning Process - Session 2 (2024-07-10, 17:32). Continued observation of the patient's spontaneous breathing capabilities later in the same day, demonstrating sustained respiratory stability without mechanical support.
- **Video S8:** Ventilator Weaning Process - Session 3 (2024-07-23, 05:10). Early morning assessment of the patient's ability to remain off the ventilator, indicating long-term improvement in respiratory muscle function.
- **Video S9:** Ventilator Weaning Process - Session 4 (2024-07-23, 20:38). Evening monitoring of the patient's spontaneous breathing status, further confirming the effectiveness of the intervention on respiratory recovery.
- **Video S10:** Ventilator Weaning Process - Session 5 (2024-07-27, 11:01). Documentation of the patient's stable respiratory status during the weaning process, showing reduced dependence on mechanical ventilation.
- **Video S11:** Ventilator Weaning Process - Session 6 (2024-07-27, 15:56). Final documented session in this series showing the patient successfully maintaining spontaneous breathing, highlighting significant recovery from the critical state.
- **Video S12:** Rehabilitation and Nursing: Body Position Changes (2024-07-04). Video demonstrating the assisted body position changes for the patient to prevent pressure ulcers and improve comfort, reflecting the patient's physical status during the recovery phase.
- **Video S13:** Baseline Physical Status: Body Position Changes (2023-10-09). Archived footage from October 2023 showing the patient's physical condition and the nursing care required for body position changes prior to the current intervention period.
