# Supplementary material for: Case Report: Herbal nutritional intervention in a critically ill ALS patient with multidrug-resistant infection
Source: Front Med (Lausanne). 2026 Oct 7;13:1912856. doi: 10.3389/fmed.2026.1912856 (PMC13683183; doi:10.3389/fmed.2026.1912856)
Supplement: Supplementary file 4 [file Table1.docx]

Table S1 The comprehensive lists of all botanical ingredients for both beverages

| Formulation | Latin Binomial (Scientific Name) | Pinyin / Common English Name |
| --- | --- | --- |
| SSPGY | *Morus alba fruit*  *Taraxacum mongolicum*  *Zingiber officinale*  *Mel*  *Momordica charantia*  *Stigma Maydis*  *Lonicera japonica*  *Imperata cylindrica*  *Capsicum annuum*  *Solanum lycopersicum*  *Malus domestica*  *Dioscorea opposita*  *Citrus reticulata*  *Siraitia grosvenorii*  *Dimocarpus longan*  *Crataegus pinnatifida*  *Pueraria lobata*  *Ziziphus jujuba*  *Vigna umbellata*  *Mentha haplocalyx*  *Nelumbo nucifera*  *Polygonatum odoratum*  *Morus alba leaves*  *Ficus hirta*  *Houttuynia cordata*  *Hylocereus undatus*  *Euryale ferox*  *Polygonatum sibiricum*  *Platycodon grandiflorus*  *Lilium brownii*  *Chrysanthemum morifolium*  *Glycyrrhiza uralensis* | Sang Shen / Mulberry fruit  Pu Gong Ying / Dandelion  Sheng Jiang / Fresh Ginger  Feng Mi / Honey  Ku Gua / Bitter melon  Yu Mi Xu / Corn silk  Jin Yin Hua / Honeysuckle flower  Bai Mao Gen / Cogongrass rhizome  Red sweet pepper  Tomato  Apple  Shan Yao / Chinese yam  Chen Pi / Tangerine peel  Luo Han Guo / Monk fruit  Long Yan Rou / Longan aril  Shan Zha / Hawthorn fruit  Ge Gen / Kudzu root  Da Zao / Jujube  Chi Xiao Dou / Rice bean  Bo He / Mint  Lian Zi / Lotus seed  Yu Zhu / Fragrant Solomonseal rhizome  Sang Ye / Mulberry leaf  Wu Zhi Mao Tao / Hairy Fig  Yu Xing Cao / Fish mint  Huo Long Guo / Dragon fruit  Qian Shi / Gordon euryale seed  Huang Jing / Solomon’s Seal rhizome  Jie Geng / Balloon flower root  Bai He / Lily bulb  Ju Hua / Chrysanthemum flower  Gan Cao / Licorice root |
| WZMTHZ | *Ficus hirta*  *Ziziphus jujuba*  *Morus alba fruit*  *Dioscorea opposita*  *Euryale ferox*  *Dimocarpus longan*  *Castanea mollissima*  *Ginkgo biloba*  *Ficus carica*  *Hylocereus undatus*  *Solanum lycopersicum*  *Malus domestica*  *Sesamum indicum*  *Pueraria lobata*  *Cannabis sativa*  *Zingiber officinale*  *Triticum aestivum*  *Lycium barbarum*  *Glycine max*  *Siraitia grosvenorii*  *Laminaria japonica*  *Chaenomeles speciosa*  *Citrus medica var. sarcodactylis*  *Poria cocos*  *Polygonatum sibiricum*  *Lilium brownii*  *Foeniculum vulgare*  *Polygonatum odoratum*  *Mel*  *Prunus mume*  *Citrus reticulata*  *Crataegus pinnatifida*  *Nelumbo nucifera*  *Glycyrrhiza uralensis*  *Cinnamomum cassia* | Wu Zhi Mao Tao / Hairy Fig  Hong Zao / Red date  Sang Shen / Mulberry fruit  Shan Yao / Chinese yam  Qian Shi / Gordon euryale seed  Long Yan Rou / Longan aril  Ban Li / Chinese chestnut  Bai Guo / Ginkgo seed  Wu Hua Guo / Fig  Huo Long Guo / Dragon fruit  Tomato  Apple  Hei Zhi Ma / Black sesame  Ge Gen / Kudzu root  Huo Ma Ren / Hemp seed  Jiang / Ginger  Xiao Mai / Wheat  Gou Qi Zi / Goji berry  Hei Dou / Black soybean  Luo Han Guo / Monk fruit  Kun Bu / Kelp  Mu Gua / Chinese quince/Papaya  Fo Shou / Buddha's hand  Fu Ling / Poria mushroom  Huang Jing / Solomon’s Seal rhizome  Bai He / Lily bulb  Xiao Hui Xiang / Fennel  Yu Zhu / Fragrant Solomonseal rhizome  Feng Mi / Honey  Wu Mei / Dark plum  Chen Pi / Tangerine peel  Shan Zha / Hawthorn fruit  Lian Zi / Lotus seed  Gan Cao / Licorice root  Rou Gui / Cinnamon bark |
