# Supplementary material for: Case Report: Herbal nutritional intervention in a critically ill ALS patient with multidrug-resistant infection
Source: Front Med (Lausanne). 2026 Oct 7;13:1912856. doi: 10.3389/fmed.2026.1912856 (PMC13683183; doi:10.3389/fmed.2026.1912856)
Supplement: Supplementary file 5 [file Table2.docx]

**Supplementary Table S2 Antimicrobial Susceptibility Testing (AST) Results for *Klebsiella pneumoniae***

| **Date (YYYY-MM-DD)** | **Specimen Type** | **Resistant (R)** | **Intermediate (I)** | **Susceptible (S)** |
| --- | --- | --- | --- | --- |
| 2023-12-28 | Sputum | Amikacin, Ampicillin, Sulbactam, Aztreonam, Cefazolin, Cefepime, Ceftazidime, Ceftriaxone, Ciprofloxacin, Doripenem, Ertapenem, Gentamicin, Imipenem, Levofloxacin, Meropenem, Minocycline, Piperacillin, Tazobactam, Tetracycline, Ticarcillin/clavulanic acid, Tobramycin, Trimethoprim/sulfamethoxazole | None | Tigecycline |
| 2024-01-02 | BALF | Doxycycline, Ampicillin, Ampicillin/sulbactam, Piperacillin/tazobactam, Cefoperazone/sulbactam, Ticarcillin/clavulanic acid, Cefazolin, Cefuroxime, Ceftriaxone, Ceftazidime, Cefepime, Aztreonam, Imipenem, Meropenem, Tobramycin, Amikacin, Levofloxacin, Ciprofloxacin, Trimethoprim/sulfamethoxazole, Minocycline | None | Tigecycline, Colistin |
| 2024-01-05 | BALF | Doxycycline, Ampicillin, Ampicillin/sulbactam, Piperacillin/tazobactam, Cefoperazone/sulbactam, Ticarcillin/clavulanic acid, Cefazolin, Cefuroxime, Ceftriaxone, Ceftazidime, Cefepime, Aztreonam, Imipenem, Meropenem, Tobramycin, Amikacin, Levofloxacin, Ciprofloxacin, Trimethoprim/sulfamethoxazole, Minocycline | None | Tigecycline, Colistin |
| 2024-01-09 | BALF | Doxycycline, Ampicillin, Ampicillin/sulbactam, Piperacillin/tazobactam, Cefoperazone/sulbactam, Ticarcillin/clavulanic acid, Cefazolin, Cefuroxime, Ceftriaxone, Ceftazidime, Cefepime, Aztreonam, Imipenem, Meropenem, Tobramycin, Amikacin, Levofloxacin, Ciprofloxacin, Trimethoprim/sulfamethoxazole, Minocycline | None | Tigecycline, Colistin |
| 2024-01-15 | BALF | Doxycycline, Ampicillin, Colistin, Ampicillin/sulbactam, Piperacillin/tazobactam, Cefoperazone/sulbactam, Ticarcillin/clavulanic acid, Cefazolin, Cefuroxime, Ceftriaxone, Ceftazidime, Cefepime, Aztreonam, Imipenem, Meropenem, Tobramycin, Amikacin, Levofloxacin, Ciprofloxacin, Trimethoprim/sulfamethoxazole, Minocycline | None | Tigecycline |
| 2024-01-22 | Sputum | Gentamicin, Cefazolin, Tobramycin, Amikacin, Amoxicillin/clavulanic acid, Ampicillin/sulbactam, Ertapenem, Trimethoprim/sulfamethoxazole, Ciprofloxacin, Meropenem, Piperacillin/tazobactam, Cefepime, Cefuroxime, Cefotaxime, Cefoxitin, Imipenem, Levofloxacin, Aztreonam, Ceftazidime, Moxifloxacin, Cefoperazone/sulbactam, Minocycline, Ticarcillin/clavulanic acid | Polymyxin B | Chloramphenicol, Tigecycline |
| 2024-01-22 | Urine | Gentamicin, Cefazolin, Tobramycin, Amikacin, Amoxicillin/clavulanic acid, Ampicillin/sulbactam, Ertapenem, Trimethoprim/sulfamethoxazole, Ciprofloxacin, Meropenem, Piperacillin/tazobactam, Cefepime, Cefuroxime, Cefotaxime, Cefoxitin, Imipenem, Levofloxacin, Aztreonam, Ceftazidime, Moxifloxacin, Cefoperazone/sulbactam, Minocycline, Ticarcillin/clavulanic acid, Nitrofurantoin | Polymyxin B | Tigecycline |
| 2024-03-01 | Sputum | Gentamicin, Cefazolin, Tobramycin, Amikacin, Amoxicillin/clavulanic acid, Ampicillin/sulbactam, Ertapenem, Trimethoprim/sulfamethoxazole, Ciprofloxacin, Meropenem, Piperacillin/tazobactam, Cefepime, Cefuroxime, Cefotaxime, Cefoxitin, Imipenem, Levofloxacin, Aztreonam, Ceftazidime, Moxifloxacin, Cefoperazone/sulbactam, Minocycline, Ticarcillin/clavulanic acid | Chloramphenicol, Polymyxin B | Tigecycline |

*BALF: Bronchoalveolar lavage fluid.

Note: In the report dated 2023-12-28, nitrofurantoin (MIC >64 μg/mL) was reported as having no breakpoint and was therefore not categorized as resistant, intermediate, or susceptible.
